# Exosomes-based dual drug-loaded nanocarrier for targeted and multiple proliferative vitreoretinopathy therapy

**DOI:** 10.1093/rb/rbae081

**Published:** 2024-06-29

**Authors:** Peiyi Zhao, Jiahao Wang, Huiying Huang, Zhirong Chen, Hui Wang, Quankui Lin

**Affiliations:** National Engineering Research Center of Ophthalmology and Optometry, Department of Biomaterials, School of Biomedical Engineering, School of Ophthalmology and Optometry, Eye Hospital, Wenzhou Medical University, Wenzhou 325027, China; National Engineering Research Center of Ophthalmology and Optometry, Department of Biomaterials, School of Biomedical Engineering, School of Ophthalmology and Optometry, Eye Hospital, Wenzhou Medical University, Wenzhou 325027, China; National Engineering Research Center of Ophthalmology and Optometry, Department of Biomaterials, School of Biomedical Engineering, School of Ophthalmology and Optometry, Eye Hospital, Wenzhou Medical University, Wenzhou 325027, China; National Engineering Research Center of Ophthalmology and Optometry, Department of Biomaterials, School of Biomedical Engineering, School of Ophthalmology and Optometry, Eye Hospital, Wenzhou Medical University, Wenzhou 325027, China; National Engineering Research Center of Ophthalmology and Optometry, Department of Biomaterials, School of Biomedical Engineering, School of Ophthalmology and Optometry, Eye Hospital, Wenzhou Medical University, Wenzhou 325027, China; National Engineering Research Center of Ophthalmology and Optometry, Department of Biomaterials, School of Biomedical Engineering, School of Ophthalmology and Optometry, Eye Hospital, Wenzhou Medical University, Wenzhou 325027, China

**Keywords:** proliferative vitreoretinopathy, epithelial-mesenchymal transformation, retinal pigment epithelial cells, exosomes, nanomedicine

## Abstract

Proliferative vitreoretinopathy (PVR) is a common cause of vision loss after retinal reattachment surgery and ocular trauma. The key pathogenic mechanisms of PVR development include the proliferation, migration and epithelial-mesenchymal transition (EMT) of retinal pigment epithelial cells (RPEs) activated by the growth factors and cytokines after surgery. Although some drugs have been tried in PVR treatments as basic investigations, the limited efficacy remains an obstacle, which may be due to the single pharmacological action and lack of targeting. Herein, the anti-proliferative Daunorubicin and anti-inflammatory Dexamethasone were co-loaded in the RPEs-derived exosomes (Exos), obtaining an Exos-based dual drug-loaded nanocarrier (Exos@D-D), and used for multiple PVR therapy. Owing to the advantages of homologous Exos and the dual drug loading, Exos@D-D showed good RPEs targeting as well as improved uptake efficiency, and could inhibit the proliferation, migration, as well as EMT of RPEs effectively. The animal studies have also demonstrated that Exos@D-D effectively inhibits the production of proliferative membranes and prevents the further development of inflammation, shows significant therapeutic effects on PVR and good biocompatibility. Such Exos-based dual drug-loaded nanocarrier investigation not only provides a promising approach for multifunctional exosome drug delivery systems construction, but also has great potential in PVR clinical therapy application.

## Graphical abstract

**Figure:**
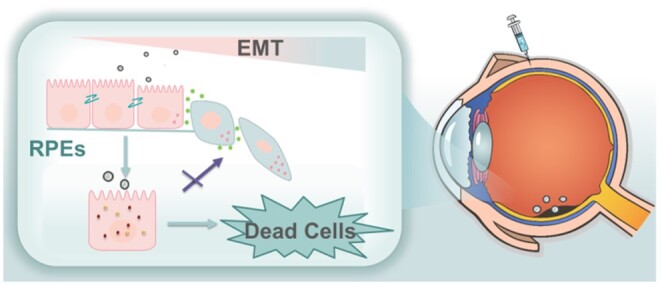

## Introduction

Proliferative vitreoretinopathy (PVR) is a blinding eye disease with abnormal scarring, which may due to the excessive wound healing lead to irreversible fibrosis in the eye [1, 2]. It is a serious complication that occurs during the natural course of rhegmatogenous retinal detachment (RD) or after RD surgery [3]. The typical disease symptom of PVR is the formation of avascular fibrotic membranes at the anterior or posterior retina in the vitreous cavity while shrinking of the fibrous membranes may further result in retractive RD [4, 5]. It is reported that PVR occurs in 5–11% of RD patients [6–8]. In addition, PVR also has a high incidence in high myopia and elderly populations [9]. Currently, the main treatment for PVR is surgery. However, the postoperative cell proliferations of the retinal pigment epithelial cells (RPEs) remain at a high recurrence rate. At present, there is no pharmacological intervention has been applied in PVR therapy, which may due to the effectiveness and biosafety concerns [10–12]. Thus, effective and targeting pharmacological intervention for PVR therapy is critical.

The development of PVR can be divided into three phases: inflammatory, proliferative and scar contraction. The proliferation and migration of RPEs is the primary pathological process in PVR [13]. Normally RPEs are in the static state. However, after retinal tear or detachment, the blood–retinal barrier (BRB) is disrupted and RPEs undergo epithelial-mesenchymal transition (EMT) stimulated by a variety of growth factors and cytokines, such as transforming growth factor-β (TGF-β) and tumor necrosis factor-α (TNF-α). This is also considered as the underlying mechanism of fibrous proliferative membrane formation [14–16]. EMT is the epithelial cells lose their epithelial character and transformed into mesenchymal cells when subjected to particular stimuli [17]. During this process, RPEs acquire a high degree of invasiveness, and resistance to apoptosis and lead to the irregular deposition of extracellular matrix proteins, including fibronectin (FN) and α-smooth muscle actin (α-SMA) [18]. TGF-β1 is an important growth factor, which is heavily upregulated when EMT and pathological fibrosis happen in RPEs [19–21]. Since PVR development is a combined effect of the inflammation and cell proliferation, the targeted multiple inventions may be better in PVR development inhibiting.

Daunorubicin hydrochloride (DNR) is an anthracycline antibiotic that blocks cell proliferation by several different mechanisms, which has been reported to have anti-proliferative effects on PVR in studies [22–25]. Dexamethasone sodium phosphate (DEX-SP) is a synthetic corticosteroid that has anti-inflammatory and immunosuppressive effects [26–28]. It also can inhibit extracellular matrix deposition, prevent adhesions and scar formation and inhibit mitosis at high doses [29–32]. The intravitreous injection of dexamethasone to inhibit PVR is considered as a promising treatment as it can effectively reduce post-vitrectomy complication occurrences [33]. Thus the combination of these two drugs in PVR treatment may achieve better therapeutic results.

**Figure 1.**
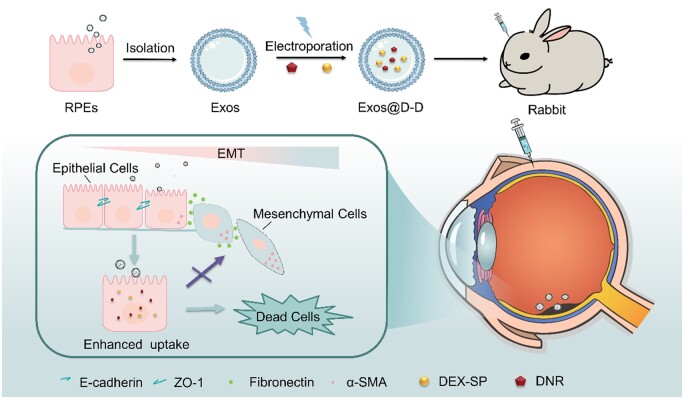
Schematic illustration of the exos@D-D construction and its inhibiting effects on the proliferation, migration and EMT of RPEs and PVR development.

On the other hand, exosomes (Exos) have received considerable attention in drug carrier research in recent years due to their low immunogenicity, high biocompatibility and strong targeting properties [34–38]. Exos are extracellular vesicles (EVs) with diameters of 40–150 nm [39–41]. They contain proteins and lipids from source cells and can freely pass through physiological barriers, participating in intercellular substance exchange and communications [42–46]. What’s more, they can be served as drug nanocarriers and capable of loading various drugs in it. Exos contain trans-membrane proteins that enhance cellular endocytosis, thus facilitating the delivery of their internal contents and reducing drug loss during transport [47–51]. Exos of different cellular origins contain specific membrane proteins and can selectively act on recipient cells to improve therapeutic efficacy [52]. Previous research has shown that Exos derived from residual lens epithelial cells (LECs) have good homologous targeting to LEC. Given this, this study used RPEs-derived Exos as drug carriers to co-load DEX-SP and DNR for the treatment of PVR (Figure 1). The dual drug-loaded Exos (Exos@D-D) was prepared by encapsulating DEX-SP and DNR into RPEs-derived Exos by electroporation. The *in vitro* RPEs as well as the *in vivo* rabbit models were established to investigate the inhibitory effects of Exos@D-D on cell proliferation, migration, EMT, as well as PVR development.

## Materials and methods

### Reagents and antibodies

DNR and Doxorubicin hydrochloride (DOX) were purchased from Meilunbio. DEX-SP was purchased from Sigma-Aldrich. Human Recombinant Transforming Growth Factor β1 (TGFβ-1) was purchased from Sino Biological. 0.05% Trypsin-EDTA, Fetal Bovine Serum (FBS), Penicillin-Streptomycin solution, Dulbecco’s modified eagle’s medium and Ham’s F12 medium (DMEM/F12) cell culture media and other cell culture-related reagents were purchased from Gibco. Calcein/PI Cell Viability/Cytotoxicity Assay Kit, Hoechst 33342, Cell Counting Kit-8 (CCK-8), 1,1'-dioctadecyl-3,3,3',3'-tetramethylindocarbocyanine perchlorate (DiI), Enhanced BCA Protein Assay (BCA) Kit, 4% paraformaldehyde fix solution, antifade Mounting Medium with DAPI, 3, 30-dioctadecyloxacarbocyanine perchlorate (DiO) and RIPA Lysis buffer were purchased from Beyotime Biotechnology Co. Phosphate-buffered saline (PBS) was purchased from Boster Biological Technology. Exo-Quick Precipitation was purchased from SBI Precipitant. Antibodies (MAC, CD9, FN, E-cadherin, α-SMA, ZO-1, GAPDH) for western blotting (WB) and immunofluorescence (IF) were purchased from Santa Cruz. TNF-α was purchased from Affinity Biosciences.

### Cell culture and animals

Human retinal pigment epithelial cells (ARPE-19, RPEs), human corneal epithelial cells (HEC-T, HCECs) and human lens epithelial cells (HLE B3, HLECs) were originated from ATCC. The cells were cultured in DMEM/F12 complete medium under the standard cell culture procedures at 37°C in 5% CO_2_. The complete medium contained penicillin–streptomycin solution and 10% fetal bovine serum.

Two-month-old chinchilla rabbits were purchased in The Experimental Animal Centre at Wenzhou Medical University. Animal experiments were approved by the Experimental Animal Ethics Committee of Wenzhou Medical University (Approval number: wydw2021-0044).

### Isolation and purification of exosomes

RPEs-derived Exos were isolated using differential centrifugation in an ultra-high-speed centrifuge according to the standard protocols [53, 54]. Briefly, RPEs were cultured in large quantities and washed by PBS buffer (pH = 7.4) five times when they reached 90% confusion, followed by 6 ml preheated (37°C) phenol red-free culture medium (collection solution) addition and incubated for another 24 h. Then the collection solution was transferred into a centrifuge tube and centrifuged at 0.5 × 10^3^ g for 10 min and 3 × 10^3^ g for 20 min to remove cell debris and apoptotic vesicles from the supernatant. The supernatant was then filtered through a 0.22-μm microporous membrane filter (SLGP033RB, Millipore). Finally, the filtered collection solution was transferred to an Optiseal tube (Beckman Coulter) and centrifuged through an ultra-high speed frozen centrifuge (Beckman Coulter) at 1.4 × 10^5^ g for 2 h at 4°C. After centrifugation, the supernatant was discarded. The purified Exos precipitant was resuspended in an equal amount of PBS and stored in a −80°C refrigerator.

### Construction and characterization of Exos@D-D

DEX-SP and DNR were co-loaded into the Exos by electroporation by Gene electroporation instrument (Bio-Rad) to obtain Exos-based dual drug-loaded nanocarriers (Exos@D-D). Using dynamic light scattering, the size distribution and zeta potential of Exos and Exos@D-D were examined (DLS, Malvern Instrument Ltd, Malvern, UK). The morphology of Exos and Exos@D-D was observed by a scanning electron microscope (SEM, Thermo Scientific, Netherlands) [55, 56]. WB was carried out under standard protocol to examine the exosomal markers CD9 and Mac. To further confirm the successful drug loading, the characteristic absorbance of DEX-SP, DNR, Exos and Exos@D-D at 242 and 495 nm were analyzed by ultraviolet-visible spectrophotometer (UV-Vis, UV-1780, Shimadzu, Japan). The confocal laser scanning microscope (LSM 880, Zeiss, G) was used to observe the co-localization of drug-loaded Exos on the RPEs. The detailed descriptions of these experiments were shown in the support information (S2.4 Construction and Characterization of Exos@D-D).

### *In vitro* experiments

#### Uptake and targeting of Exos and Exos@D-D

To assess the uptaking and the homologous targeting properties of Exos by the RPEs, the fluorescence images were taken and analyzed by laser confocal microscopy. Briefly, the RPEs were co-cultured with equal amounts of DiO-labeled Exos for 3 or 6 h. In order to observe the difference in uptaking between drug-loaded Exos and free Exos, the autofluorescent DOX was used instead of the free drugs. Similarly, the free DOX or DOX-loaded DIO-labeled Exos was then incubated with RPEs for 6 h, followed by the above steps until assayed.

To further understand the homologous targeting of RPEs derived Exos to RPEs *in vitro*, HCECs, HLECs and RPEs were seeded into 24-well cell culture plate for 24 h. The cells were dealt with as described above cell uptaking assay until the fluorescent images were taken.

#### Anti-proliferation and migration analysis of free drug *in vitro*

The anti-proliferative ability of the free drug (DNR: 0.01, 0.05, 0.1, 0.5, 1, 10 μg/ml; DEX-SP: 0.2, 0.8, 3, 5, 10 mg/ml) was verified by CCK-8 and Hoechst 33342 staining [57, 58]. To examine its impact on cell migration, cells were treated with different concentrations of DEX-SP (0.2, 0.4, 0.8, 1 and 2 mg/ml) for 0, 24 h and 48 h, respectively, and tested by wound-healing assay. The scratch area was photographed with an inverted fluorescence microscope (DMi8, Leica, Germany). The detailed descriptions of these experiments were shown in the support information (S2.5.2 Anti-proliferation and migration analysis of free drug in vitro).

#### Establishment of cell EMT model

RPEs were grown in a 96-well cell culture plate at a density of 5 × 10^3^ cells per well under serum-free medium condition and cultured for 12 h. Then they were incubated with different concentrations of TGF-β1 (0.5, 2.5, 10 and 12.5 ng/ml) for 48 h and the cell viability was obtained by CCK-8 kit. Five culture wells were replicated at the same concentration and at the same time point. To observe changes in cell morphology, cells were incubated for 48 h with different concentrations of TGF-β1 and then observed under an inverted fluorescent microscope at 20× and 40× magnification, respectively.

#### Exos@D-D inhibits TGF-β1-induced EMT in RPEs

To investigate the drug effect on the EMT of RPEs, cells were firstly grown on a 24-well cell culture plate at a density of 6 × 10^5^ cells per well and cultured for 24 h, followed by serum-free medium replacement and starved for another 12 h. Then they were divided into four groups as follows: (i) Cells without any treatment (control group). (ii) Cells treated with 10 ng/ml TGF-β1 only. (iii) Cells treated with 10 ng/ml TGF-β1 and free drugs D-D (1 μg/ml DNR and 0.8 mg/ml DEX-SP, final concentration in the culture medium). (iv) Cells treated with 10 ng/ml TGF-β1 and Exos@D-D (20 μL Exos@D-D was added to 80 μl culture medium). Briefly, the above cultured cells were scratched by the tips to create wounds and pretreated the cells with the drug for 2 h followed by the TGF-β1 stimulation. When in the groups of the no drug treatment, the drug pretreatment procedure was omitted. Images were captured by inverted phase contrast microscopy at 0 and 48 h post-scratch to assess cell migration. The wound area was measured using Image J software (National Institutes of Health, USA). Cell migration rate was calculated as follows: cell migration rate = (original scratch width − current scratch width)/original scratch width × 100%. Three or more randomly selected fields were evaluated [59]. The cell viabilities of RPEs under different treatment conditions were detected by the Calcein/PI Cell Activity and Cytotoxicity Assay Kit after 48 h by inverted fluorescent microscope and analysis by Image J software [60].

IF analysis: RPEs were seeded and cultured in a 24-well plate inlaid with cell slides. After 48 h of drug treatment, the cells were washed three times with PBS, fixed in 4% paraformaldehyde for 10 min, and then permeabilized in 0.3% Triton X-100 and blocked with 5% BSA. Afterward, the cells were co-incubated with α-SMA, FN, ZO-1 and E-cadherin (Ecad) antibodies and washed three times with PBS at 4°C overnight. After that, cells were treated with Alexa Fluor 594-conjugated secondary antibody for 2 h. Then the Antifade Mounting Medium with DAPI was added. Images were subsequently captured by a fluorescence microscope.

WB experiments were first performed by treating cells treated with different drugs with RIPA Lysis buffer. Then centrifuge at 1.2 × 10^4^ rpm for 20 min at 4°C and collect the supernatant. Subsequently, the same WB procedure as above was performed. The strips were incubated with primary antibodies against FN, α-SMA, Ecad and GAPDH at 4°C overnight. After that, the matching secondary antibodies were incubated for 1 h. Lastly, the Fluor Chem E Multifunctional Imaging System was used to visualize the proteins.

### *In vivo* experiments

#### The rabbit model of PVR

An animal model of PVR was established for 2.5–3 kg chinchilla rabbits. Four groups were created at random: one group was the blank control group (Control), one group was the model group (RPEs), one group was the free dual drug treatment group (D-D) and the other group was the dual drug-loaded Exos treatment group (Exos@D-D). RPEs (1 × 10^6^/ml, 200 μl) and PBS with or without the drug (20 μl) were injected intravitreously on Day 1 of the experiment. All procedures were performed under anesthesia. The fundus was examined on preoperative Day 1, postoperative Day 1, 3, 7, 14 and 28 by using a digital fundus camera (TRC-50DX, Topcon, Japan) and an optical correlation tomography scanner OCT (Heidelberg Engineering, Heidelberg, Germany).

#### Histological and immunofluorescence analysis

The rabbits were euthanized on Day 28 and the eyes were subsequently separated and fixed. After sectioning, some samples were stained with hematoxylin-eosin (H&E) and some were used in IF analysis. The procedure of the IF analysis of the *in vivo* tissue samples was the same with that of the above *in vitro* cell samples. Fluorescence microscope (DM4B, Leica, Germany) and confocal laser scanning microscope (LSM 880, Zeiss, Germany) were used to obtain the images [61].

## Results

### Construction and characterization of Exos@D-D

**Figure 2.**
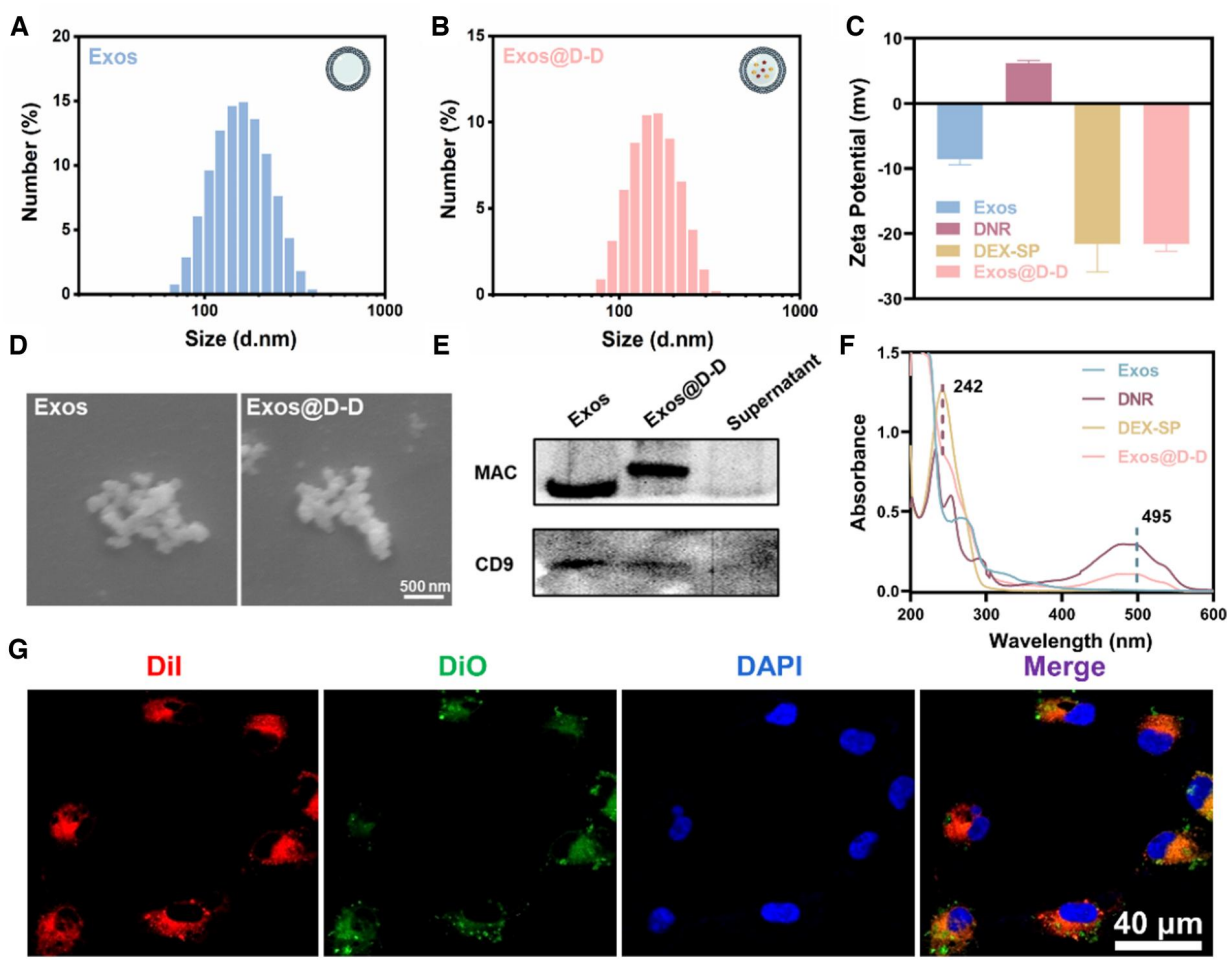
(**A**, **B**) Particle size and distribution of Exos (A) and exos@D-D (B). (**C**) Zeta potential of Exos and Exos@D-D. (**D**) Representative SEM images of Exos and Exos@D-D. (**E**) WB results showed Exos’s particular biomarker expression (CD9 and Mac) in each group that included supernatant, Exos@D-D and Exos. (**F**) UV–vis spectra of Exos, DNR, DEX-SP and Exos@D-D dispersed in PBS. (**G**) Fluorescence co-localization analysis of DiO-labeled Exos@D-D.

As shown in Figure 2A, the diameter of the isolated Exos is around 148 nm and exhibited a narrow size distribution. After drug loading, the diameter is increased (Figure 2B). It showed that the hydrodynamic diameter of Exos@D-D is around 162 nm with a narrow distribution. It can be also seen that Exos were negatively charged with a Zeta potential of −8.84 mV (Figure 2C), while the surface charge of Exos@D-D decreased in potential due to the drug loading (−21.6 mV, Figure 2C). The SEM images in Figure 2D showed that the Exos and Exos@D-D were visible as vesicle-like structures due to the structural properties of the membrane. Figure 2D showed that the exosomal markers, Mac and CD9, were clearly displayed both on Exos and Exos@D-D, but they are absent in the cell supernatant. Thus the exosomal nanoscale vesicle structure and surface functional proteins were still present in Exos@D-D. UV-Vis absorbances of free Exos, DEX, DNR and Exos@D-D samples (Figure 2F) showed the existence of characteristic absorbance peaks of drugs in Exos@D-D, which further confirmed the successful loading of DNR and DEX-SP into Exos. In addition, the fluorescence image in Figure 2G showed that DiO-labeled Exos@D-D entered the cytoplasm of RPEs, indicating that the drug-loaded Exos could be well taken up by RPEs.

### *In vitro* therapeutic effects

#### Exos and Exos@D-D uptake and targeting

**Figure 3.**
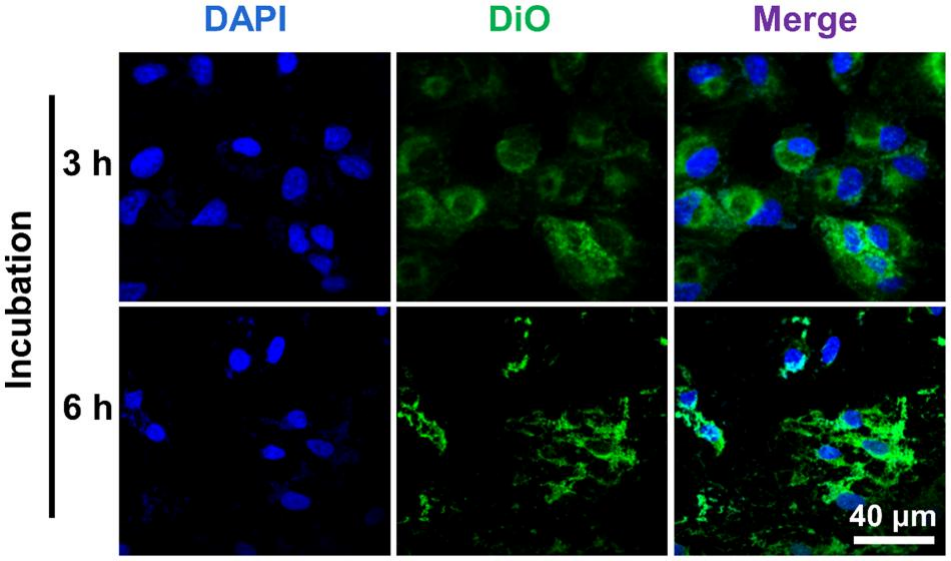
Representative fluorescence microscopy images of RPEs with DAPI-labeled nuclei after co-culturing with Exos for 3 h and 6 h, respectively.

**Figure 4.**
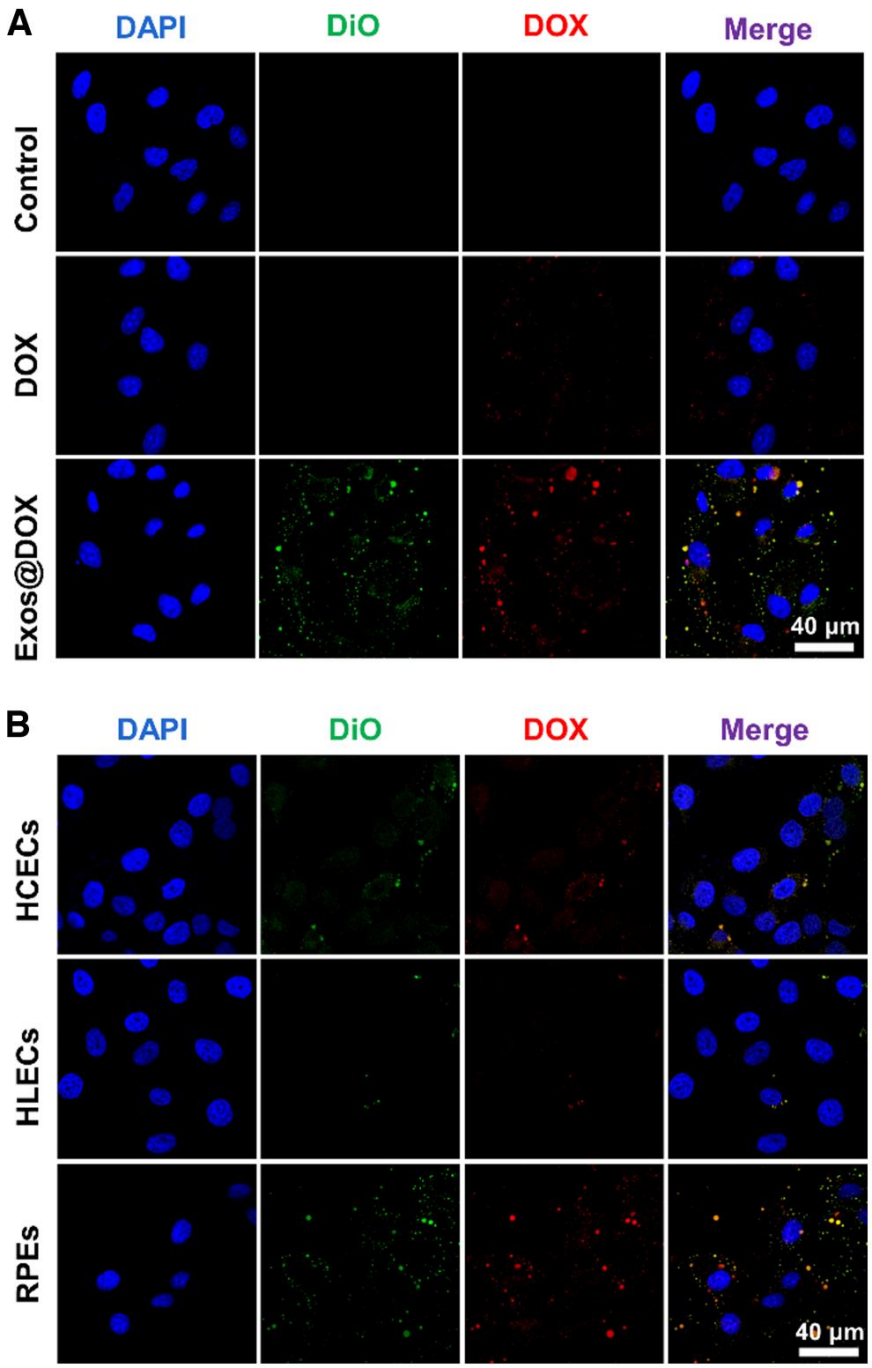
Uptake and homologous targeting analysis of drug-loaded Exos. (**A**) Representative fluorescence microscopy images of RPEs in co-culture with exos@DOX and free DOX for 6 h. (**B**) Representative fluorescence microscopy images of HCECs, HLECs and RPEs in coincubation with Exos@DOX for 6 h.

Cellular uptake capacity is essential to the efficiency of drug delivery. According to previous publications, the longer the uptake time, the more Exos are taken up by cells within a certain time frame, and there is homologous targeting between different sources of Exos [62]. Herein, the uptake of RPEs-originated DiO-labeled Exos was investigated by the co-incubation Exos with RPEs. As shown in Figure 3, the Exos can be internalized by the RPEs as short as 3 h when observation. And the internalization is more visible when the co-incubation time increases. Also, the internalization of the free DOX and Exos@DOX containing the same concentration of DOX by the RPEs were compared. The DOX and DiO-labelled Exos can be tracked by confocal laser scanning microscope and show red and green fluorescence, respectively. As shown in Figure 4A, after 6 h incubation with RPEs, the Exos@DOX group had more yellow dots (the overlap of red and green fluorescence), whereas few were shown in the DOX and Exos groups, which indicates a higher internalization rate of Exos@DOX than free DOX. The enhanced internalization may be due to the homologous targeting of the Exos. Further, the homologous targeted internalization of the Exos@DOX was also investigated by the co-incubation with the different ocular cells, including RPEs, HLECs and HCECs. As shown in Figure 4B, it can be observed that more drug-loaded Exos were taken up by RPEs than the other two cells at the same incubation time. These results suggest that Exos exhibit specific targeting behavior towards RPEs. Homologous Exos hence have higher drug delivery effectiveness and a better affinity for their parent cells.

#### In vitro anti-proliferation and migration analysis

**Figure 5.**
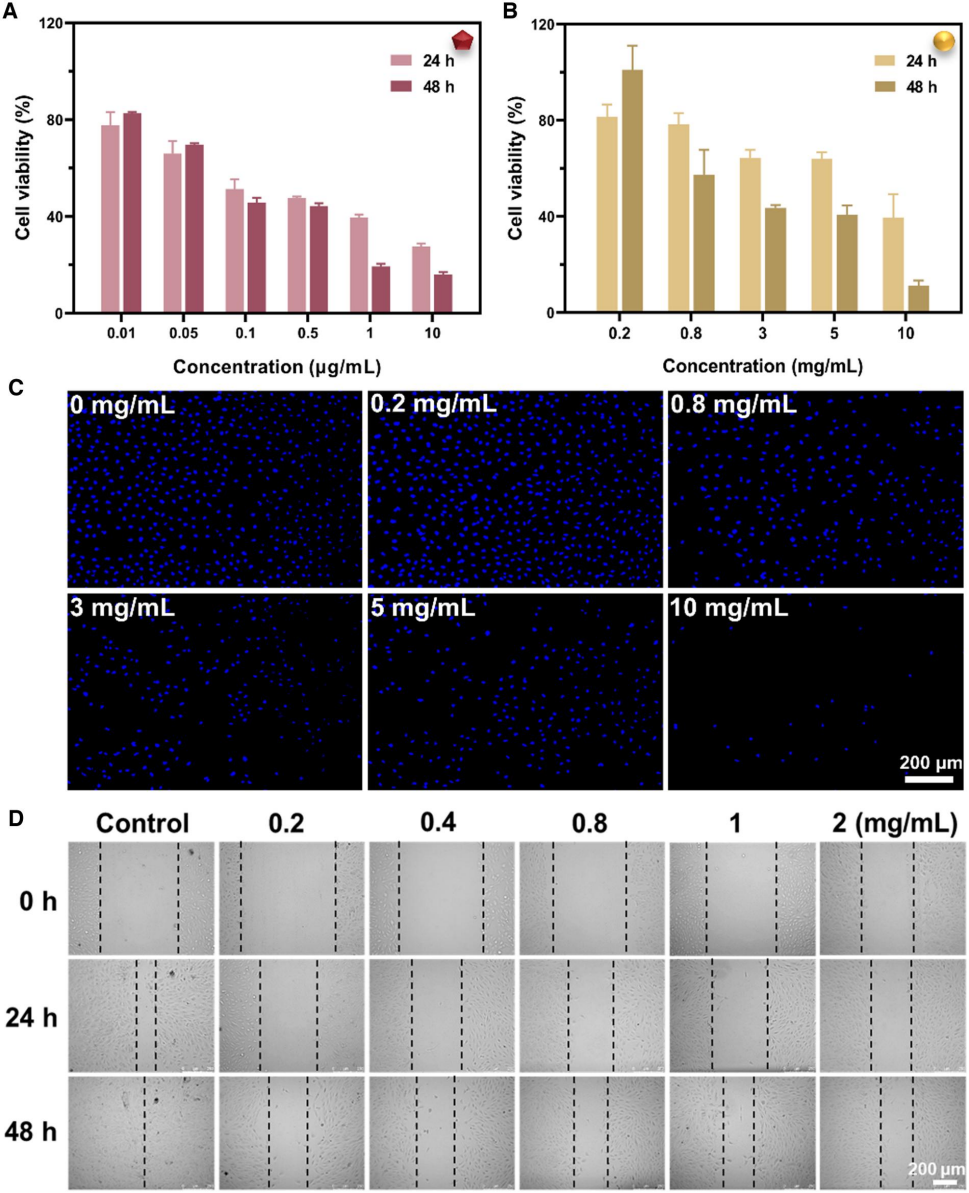
Inhibition of cell migration and proliferation effects after treated by the free drugs. (**A**, **B**) CCK-8 results of RPEs treated with different concentrations of DNR (A) and DEX-SP (B) for 24 h, 48 h. (**C**) Representative fluorescence micrographs of RPEs treated with different concentrations of DEX-SP for 48 h, Hoechst 33342-labeled nuclei. (**D**) Influence of varying DEX-SP concentrations on RPE migration.

The optimization of the drug concentration is crucial for its effective anti-proliferative and anti-migratory effects. The anti-proliferative effects of DNR and DEX-SP were investigated by the CCK-8 kit. As shown in Figure 5A and B, the cell viability decreased gradually with the drug concentrations increasing, which indicates the antiproliferative effect to the RPEs of the DNR and DEX-SP. Figure 5C shows the cell distributions after the free DEX-SP treatment via the cell nucleus stained by Hoechst 33342 staining, which also confirmed concentration-dependent anti-proliferative effect. The cell migration inhibition effect after the DEX-SP treating was also investigated by the cell scratch test. As shown in Figure 5D, it can be demonstrated that the remaining scratch area increased with increasing drug concentration, indicating that DEX-SP significantly inhibited the cell migration of RPEs whereas no morphological changes were detected. Considering the above results, the concentrations of DEX-SP at 0.8 mg/ml and DNR at 1 μg/ml were used in the subsequent experiments.

#### TGF-β1-induced EMT model of RPEs

**Figure 6.**
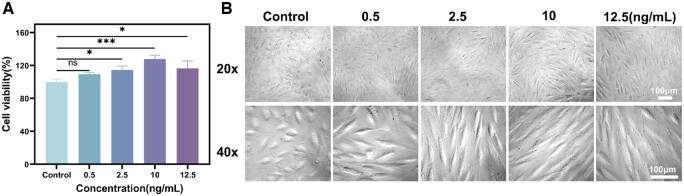
TGF-β1 induces EMT in RPEs. (**A**) CCK-8 results of RPEs treated with different concentrations of TGF-β1. *P < 0.05, ***P < 0.001. ns, not significant. (**B**) Effect of different concentrations of TGF-β1 on the morphology of RPEs.

During the EMT process, RPEs will change their morphology, polarity and migratory capacity, and undergo cytoskeletal remodeling and fibrosis. Herein, the TGF-β1 was used to induce the EMT model in the RPEs. As illustrated in Figure 6A, the cell viability was tested after TGF-β1 treatment at different concentrations. There was a statistically significant difference after 48 h incubation, with the highest level of cell proliferation observed at TGF-β1 concentration of 10.0 ng/ml. The cell morphology was also observed after TGF-β1 treatment. And as shown in Figure 6B, the morphology of RPEs changed significantly after 10.0 ng/ml TGF-β1 treatment for 48 h. It can be seen that RPEs lost their usual epithelial phenotype and their morphology gradually altered from cobblestone-like to long spindle-shaped mesenchymal-like cells. Therefore, treatment of RPEs with 10.0 ng/ml TGF-β1 for 48 h can successfully induce the EMT model of RPEs.

#### Exos@D-D inhibits TGF-β1-induced EMT in RPEs

**Figure 7.**
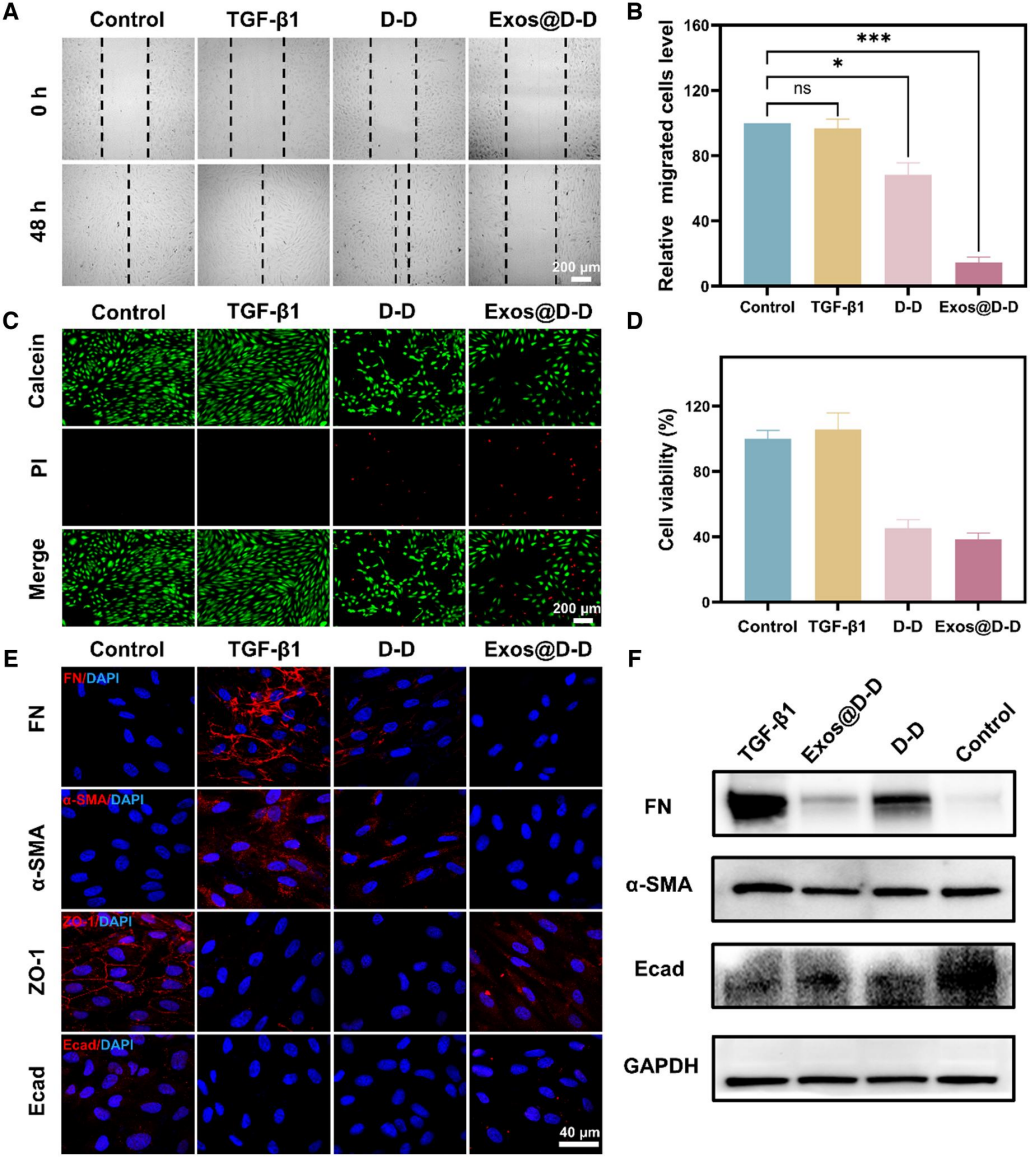
Exos@D-D inhibits TGF-β1-induced EMT in RPEs. (**A**, **B**) Wound healing assay showing the effect of different drugs on TGF-β1-induced cell migration. Data are expressed as mean ± SD, compared to untreated controls, *P < 0.05; ***P < 0.001. ns, not significant. (**C**, **D**) Calcein/PI Cell Viability/Cytotoxicity Assay Kit assay results: Exos@D-D inhibits TGF-β1-induced cell proliferation. (**E**, **F**) Results of cellular IF staining for changes in EMT markers and WB assay: Exos@D-D inhibited the upregulation of FN, and α-SMA induced by 10 ng/ml TGF-β1 in RPEs and suppressed the downregulation of ZO-1 and Ecad.

The EMT inhibition effect of Exos@D-D was investigated in the 10.0 ng/ml TGF-β1-induced EMT in RPEs. The cell migration was greatly decreased after Exos@D-D treatment. As demonstrated by Figure 7A and B, the scratch area of the RPEs in the TGF-β1 group after 48 h was almost disappeared, whereas the remaining scratch area in the other two treatment groups (drugs without or with Exos encapsulation: D-D, Exos@D-D) was remains when compared with the control group. The remaining scratch area in Exos@D-D was larger than that in D-D. Additionally, the cell migration rate was lower, suggesting that TGF-β1 effectively improved the ability of RPEs to migrate and that Exos@D-D had a better inhibitory effect on RPE migration in comparison to D-D.

In addition, the results of the Calcein/PI cell activity and cytotoxicity assay as shown in Figure 7C and D. It showed that the green fluorescence (Calcein AM staining, live cells) of the group treated with TGF-β1 alone was stronger than that of the untreated group. This result further indicated that the induction of TGF-β1 would result in cells acquiring stronger anti-proliferative ability. Exos@D-D showed less green fluorescence compared to the D-D treated group, suggesting that Exos@D-D has a better anti-proliferative effect than the free drugs. Combining the above results, the enhanced homologous targeting and internalization property of the Exos may be primarily responsible for Exos@D-D’s stronger anti-proliferative activity.

The results of the IF experiments and WB experiments are shown in Figure 7E and F. These results indicate that TGF-β1 could upregulate the EMT markers while suppressing the expression of epithelial cell markers. Compared with the group treated with TGF-β1 alone, Exos@D-D exhibited an inhibitory effect on EMT marker overexpression, inhibited the downregulation of ZO-1 and Ecad, and successfully controlled the TGF-β1-induced increase of FN and α-SMA protein expression levels. All of these results suggest that Exos@D-D can inhibit the TGF-β1-induced EMT process in RPEs.

### *In vivo* therapeutic effects

#### Observations on the efficacy of intraocular inhibition of the PVR process

**Figure 8.**
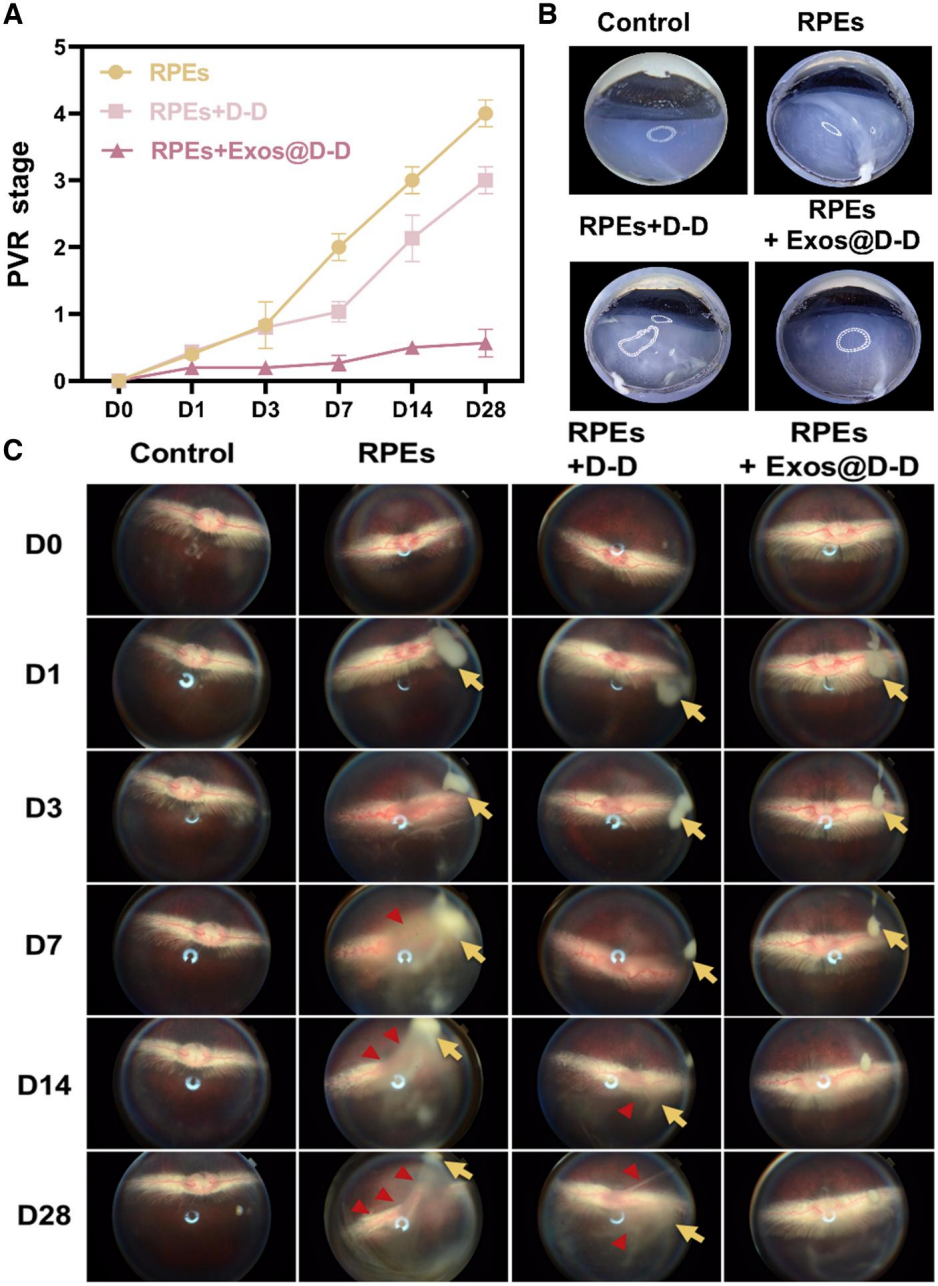
Exos@D-D inhibits the intraocular PVR process. (**A**) Grading of PVR in different drug-treated groups. (**B**) Horizontal section of the eye. (**C**) Representative fundus observation images at different periods. Long arrows represent cell proliferation and short arrows represent the production of the anterior retinal membrane.

The inhibitory effect of Exos@D-D on experimental PVR was in the PVR model of rabbits. The post-treatment effects of the different treatment groups were graded in a Fastenberg grading fashion. The results are shown in Figure 8A. It can be seen that the PVR grade of RPE+Exos@D-D treated eyes is all lower than 1 even in the Day 28, whereas the PVR grade increased gradually and obviously in other two groups, no matter RPE+D-D or RPE alone treated eye. (The PVR grading standards were shown in the support information S3.3) The findings imply that Exos@D-D can successfully impede PVR development. Then the eyes were dissected along the horizontal plane with lens be removed to observe the retinal surface. As shown in Figure 8B, the retinal surface of the model group (RPEs) produced an implicated proliferative membrane, and the retinal surface of the treatment group (D-D) had a large number of proliferative cells, while the retinal surface of the treatment group (Exos@D-D) had no significant abnormalities as in the control group.

**Figure 9.**
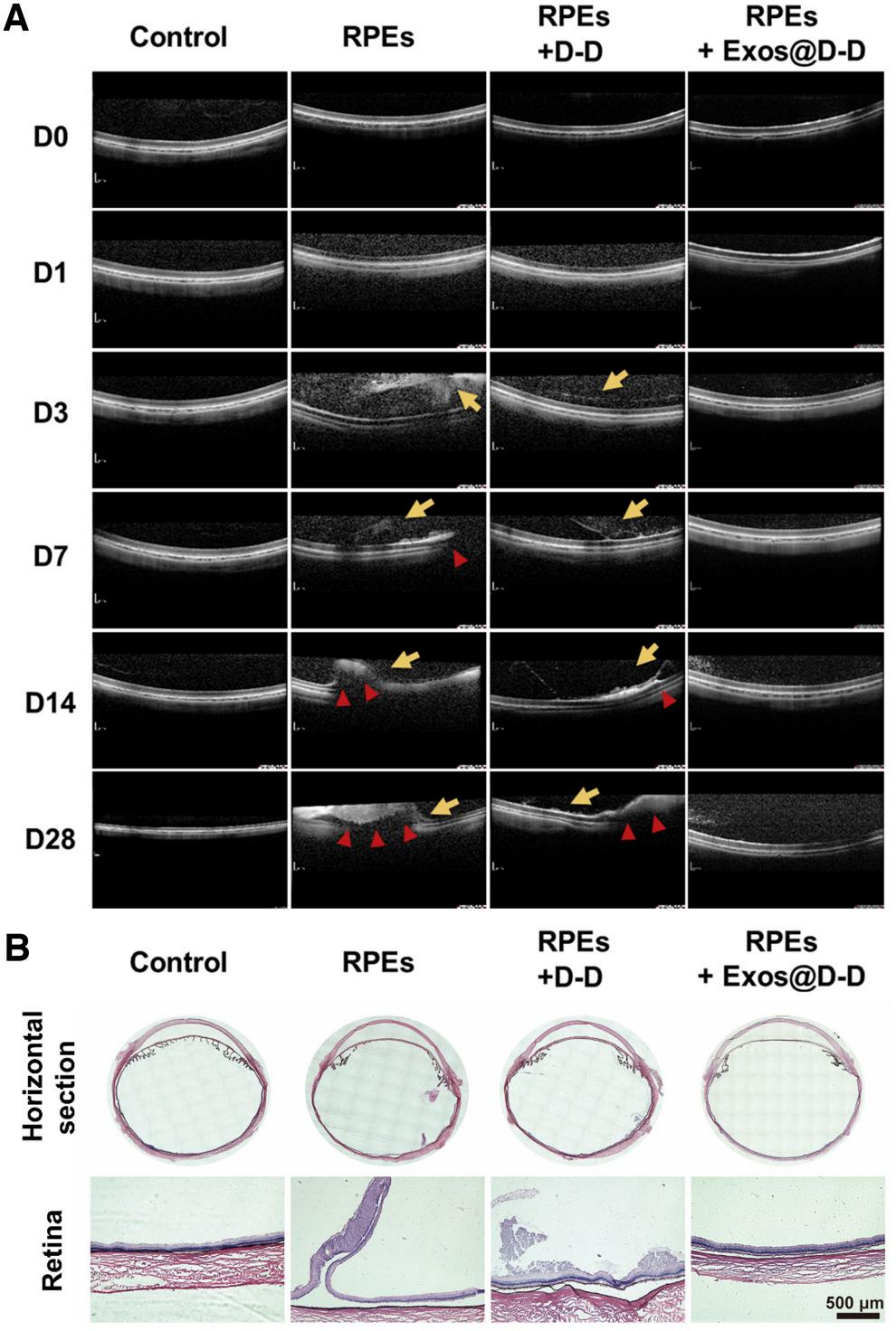
(**A**) Representative OCT observation images at different periods. Long arrows represent cell proliferation and short arrows represent the production of the retinal adventitia. (**B**) Representative (H&E) staining results for each drug treatment group.

The posterior segment of rabbit eyes was evaluated by fundus photography and the OCT imaging system pre-operation (D0) and on postoperative Days 1, 3, 7, 14 and 28. Fundus photography results are shown in Figure 8C. The yellow arrows indicated cell proliferation and red arrows indicated the production of proliferating membranes. Grade 0 PVR was observed in all control eyes. Fundus imaging showed that no abnormalities in the retina, no cells in the vitreous and the optic disc and blood vessels were visible in each group. In the treated group (Exos@D-D), the vitreous humor was relatively clear, similar to that of the control group. On Day 14, a large number of cell streaks were observed in the model group (RPEs) in addition to a large number of proliferated cells. A small number of cell streaks were also detected in the D-D treatment group, whereas the number of proliferated cells was greatly decreased in the treatment group (Exos@D-D). The production and retraction of retinal proliferative membranes with serious or mild local detachment of medullary rays were observed on Day 28 in the model group (RPEs) or D-D treated group, whereas the retina morphology was kept well in the Exos@D-D treated group. These results were also confirmed by OCT images (Figure 9A). It showed that the model group (RPEs) did produce localized RD as early as on Day 14 and it became serious on Day 28. On Day 28, a certain extent localized RD was also found in the D-D treated group, while the retina in the Exos@D-D-treated group showed no significant abnormalities. All these results indicate that the progression of PVR was notably inhibited in the treated (Exos@D-D) eyes compared to the control eyes.

An essential tool for assessing the safety of materials used in intraocular implants is pathological tissue sections [63]. Typical H&E results of each group’s horizontal eye sections and local retinal sections are shown in Figure 9B. In the treatment group (D-D), there was extensive irregular cell proliferation and retinal thickening on the retinal surface, while in the model group (RPEs), there was marked retinal adventitia and RD. The area of RD typically occurs around the optic nerve. In contrast, the treatment group (Exos@D-D) showed no obvious RD and a relatively smooth surface, all layers of the retina were normal and there was no anterior retinal membrane or vitreous constriction membrane. The cornea and iris were not significantly different from the control group, again with normal morphology and structural integrity. This result indicates that Exos@D-D infusion is safe for the eye.

#### IF analysis results

**Figure 10.**
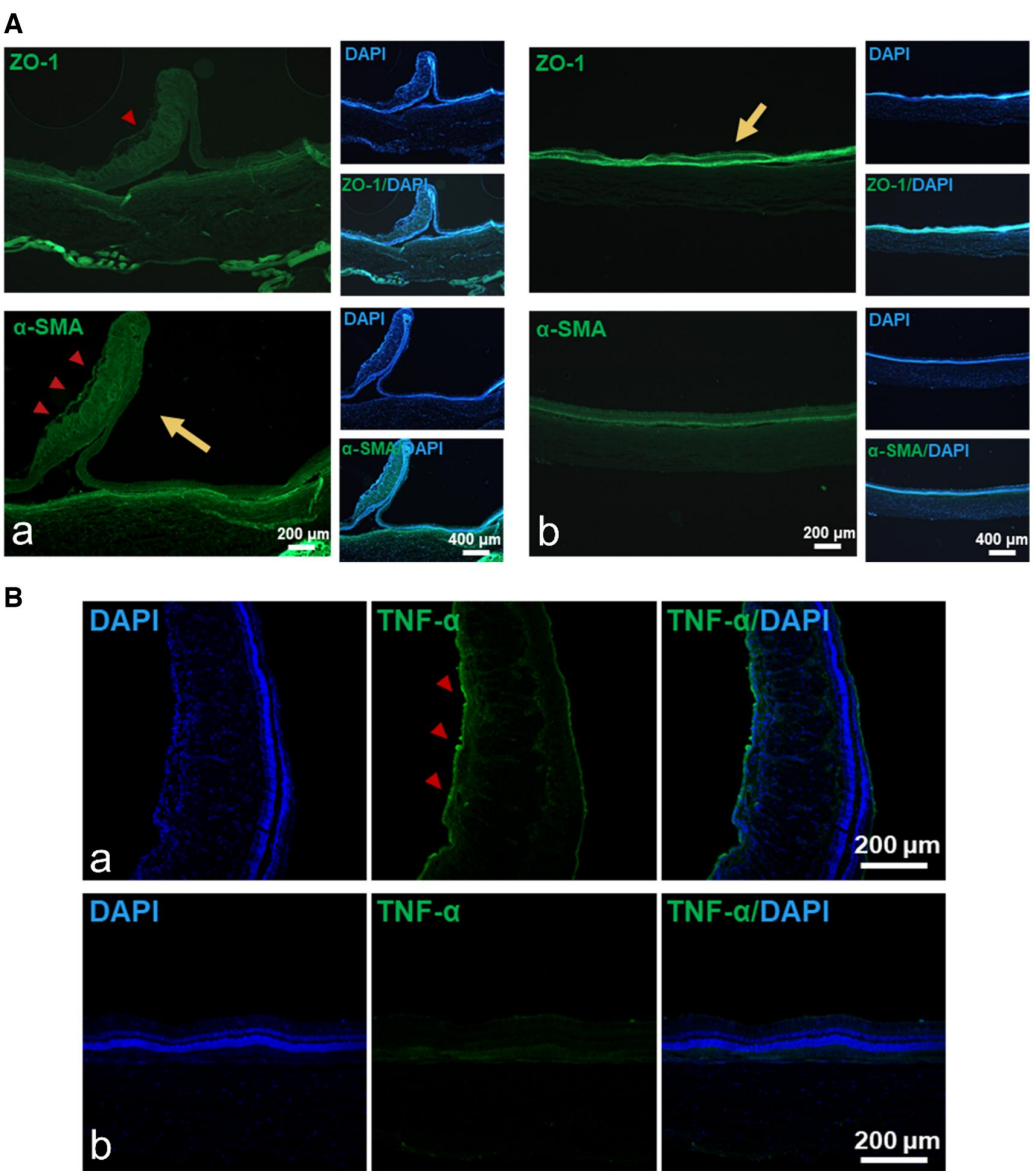
(**A**) Exos@D-D inhibits the formation of the retinal adventitia, downregulation of ZO-1, and upregulation of α-SMA. IF staining of retinal tissue in (a) model group (RPEs), (b) treatment group (Exos@D-D). The short arrows represent the generation of the retinal adventitia with an unsmooth retinal surface. Long arrows represent the upregulation of fluorescence intensity. (**B**) Exos@D-D inhibits the expression of TNF-α. IF staining of retinal tissue in (a) model group (RPEs), (b) treatment group (Exos@D-D). Short arrows indicate high expression of TNF-α and unsmooth retinal surface.

Figure 10A showed the ZO-1and α-SMA staining results in retinal tissue sections from the model group (RPEs) and treatment group (Exos@D-D). It showed that darker fluorescence intensity of ZO-1 staining and less fluorescence intensity of α-SMA staining were found in the Exos@D-D treated group than in the model group (RPEs). In the model group, loss of tight junctions between the retinal epithelial cells and a stronger fluorescence on the α-SMA staining of the distinct α-SMA-positive retinal adventitia were shown in the Figure 10A-a. In combination with the H&E-stained images (Figure 9B), the RD was more visually evident. In contrast, in the treatment group (Exos@D-D), ZO-1 staining showed that the retinal layers were tightly connected and no RD occurred, and the α-SMA staining showed no strong fluorescence and no α-SMA-positive retinal adventitia was observed (Figure 10A-b).

TNF-α is a monocyte-derived cytokine that promotes inflammation. Figure 10B shows the TNF-α expression on the characteristic retinal tissue sections of the model group (RPEs) (Figure 10B-a) and treatment group (Exos@D-D) (Figure 10B-b). It showed that the model group (RPEs) rendered stronger fluorescence. In contrast, the treatment group (Exos@D-D) did not show strong fluorescence. These results indicated that the treatment also effectively inhibited the further production of inflammation.

These findings imply that the exosomes-based dual drug-loaded nanocarrier Exos@D-D can greatly prevent the PVR development. Highlighting the importance of targeted drug delivery at the site of disease and that Exos@D-D has good therapeutic efficacy and good intraocular biocompatibility, such as the exosomes-based dual drug-loaded nanocarrier Exos@D-D offers great promise for further clinical PVR prevention application after further clinical safety and detailed mechanism investigation.

## Conclusion

In conclusion, and exosomes-based dual drug loaded nanocarrier Exos@D-D was constructed for targeted and multiple PVR therapy. Based on the homologous targeting of RPEs-derived Exos, Exos@D-D showed more effective cellular uptake and targeted delivery ability when compared with free drugs. The dual drugs loaded Exos@D-D can also effectively prevent the proliferation and migration of RPEs while inhibiting the overexpression of EMT markers, showing the effective inhibition of the *in vitro* EMT process. The animal experiments also showed that Exos@D-D exhibited significant preventive and therapeutic effects on the PVR development, further inhibited inflammation, as well as had good intraocular biocompatibility. Thus, such exosomes-based dual drug loaded nanocarrier Exos@D-D has great potential in applications in PVR prevention and treatment.

## Supplementary Material

rbae081_Supplementary_Data
