# Supplementary material for: Exosomes-based dual drug-loaded nanocarrier for targeted and multiple proliferative vitreoretinopathy therapy
Source: Regen Biomater. 2024 Jun 29;11:rbae081. doi: 10.1093/rb/rbae081 (PMC11262591; doi:10.1093/rb/rbae081)
Supplement: rbae081_Supplementary_Data [file rbae081_supplementary_data.docx]

**Supporting Information**

**Exosomes-based dual drug loaded nanocarrier for targeted and multiple proliferative vitreoretinopathy therapy**

Peiyi Zhao, Jiahao Wang, Huiying Huang, Zhirong Chen, Hui Wang, Quankui Lin*

National Engineering Research Center of Ophthalmology and Optometry, School of Biomedical Engineering, School of Ophthalmology and Optometry, Eye Hospital, Wenzhou Medical University, Wenzhou 325027, China

*Corresponding Author: linqk@wmu.edu.cn

**Materials and methods**

**S2.4 Construction and Characterization of Exos@D-D**

DEX-SP and DNR were loaded into the Exos by electroporation. 250 μL drugs mixture with 5 μg/mL DNR and 4 mg/mL DEX-SP in it were mixed with 250 μL of 4°C precooled Exos. They were then electroporated using Bio-Rad Gene electroporation equipment at 250 V and 350 μF. The suspension was placed in water at 37℃ for 30 min to allow recovery of the Exos membrane, then the Exo-Quick Precipitation (SBI precipitant, USA) was added at a ratio of 5:1 and stored in refrigerator at 4 ℃ overnight. To get rid of unloaded Drugs, discard some supernatant and centrifuge for another 30 min. Finally, the purified Exos@D-D was then resuspended in an equal volume of PBS buffer. Using dynamic light scattering, the size distribution and zeta potential of Exos and Exos@D-D were examined (DLS, Malvern Instrument Ltd, Malvern, UK). The surface morphology of Exos and Exos@D-D were observed using a scanning electron microscope (SEM, Thermo Scientific, Netherlands).

WB was used to examine the exosomal markers CD9 and Mac. The BCA protein analysis kit was used to quantify the exosomal proteins.CD9 and Mac proteins were separated on the polyvinylidene difluoride (PVDF) membranes by the sodium dodecyl sulfate-polyacrylamide gel electrophoresis (SDS-PAGE). Membranes were blocked in PBS-Tween 20 (PBST) fat-free dried milk, at 37℃ for 1 h after that incubated with their primary antibodies at 4℃ overnight. The next day, the strip was washed five times with PBST and incubated with the corresponding secondary antibody for 30 min at 4℃, followed by five more washes with PBST. A final drop of ultrasensitive chemiluminescent solution was added to the strips and the proteins were subsequently visualized using a gel imaging system (Azure C300, Azure Biosystems Inc, USA).

To further confirm the successful drug load, the absorption intensities of DEX-SP, DNR, Exos, and Exos@D-D at 242 nm and 495 nm were analyzed by UV spectrophotometer (UV-1780, Shimadzu, Japan). To observe the co-localization of drug-loaded Exos in RPEs, cells from RPEs were seeded at a density of 6 ×10^4^ cells/well in a 24-well cell culture plate with a built-in cell slide, and a complete medium was added to each well and incubated overnight. Then the serum-free medium was replaced and an equal amount of DIO-labeled Exos was added to each well and incubated for 4 h. Subsequently, the cells were fixed in 4% paraformaldehyde fixative for 10 min at room temperature, followed by washing the wells with PBS buffer and then incubating with the prepared DiI dye for 20 min. After that, the cell slides were removed and dripped with Antifade Mounting Medium with DAPI. Ultimately, a confocal laser scanning microscope (LSM 880, Zeiss, G) was used to obtain the images.

**S2.5.2 Anti-proliferation and migration analysis of free drug in vitro**

The anti-proliferative ability of the free drug was observed by CCK-8 assay and Hoechst 33342 staining. Firstly, RPEs suspensions were inoculated into 96-well cell culture plate, ensuring 100 μL of cell suspension containing 5×10^3^ cells per well, and then incubated in a cell incubator. After 24 h, discard the culture medium and add the complete medium containing different concentrations of drugs (DNR: 0.01, 0.05, 0.1, 0.5, 1, 10 μg/mL; DEX-SP: 0.2, 0.8, 3, 5, 10 mg/mL) to each well according to experimental groups. After 24 and 48 h of incubation, CCK-8 reagent was added to each well. After 2 h, absorbance values were measured at 450 nm using a microplate reader (SpectraMax M5, Molecular Devices, USA). Five replicate wells were set up at the same concentration and time point, and the DEX-SP concentration of 0 mg/mL was set as the control group, DNR, and the same. The cells treated with the same concentration of DEX-SP for 48 h were subjected to Hoechst 33342 staining, and the Hoechst 33342 was incubated with RPEs in the cell incubator for 15 min, and the cells were washed once with PBS and observed under an inverted fluorescence microscope (DMi8, Leica, Germany).

DEX-SP is commonly used in the treatment of ocular inflammatory diseases. To examine its impact on cell migration, cells were treated with different concentrations of DEX-SP (0.2, 0.4, 0.8, 1, and 2 mg/mL) for 0 h, 24 h, and 48 h, respectively, by wound-healing assay. Then morphological changes of cells and scratch areas were observed at the three time points using inverted fluorescence microscopy. The cells were first seeded in 24-well cell culture plate at a density of 6×10^4^ cells/well. After 24 h of incubation, the cells were scratched in the monolayer of confluent cells with a sterile gun tip, and then the cells were divided into six groups, one group was cultured with serum-free medium, and the other five groups were treated with drugs. After 0, 24h, and 48h of dosing, the medium was discarded and carefully washed, and then the scratch area was immediately photographed with an inverted fluorescence microscope.

The PVR stages (grade) were list as below: stage 1, intravitreal membrane; stage 2, focal traction, localized vascular changes, hyperemia, engorgement, and blood vessel elevation.; stage 3, localized detachment of the medullary ray; stage 4, extensive retinal detachment, total medullary ray detachment, and peripapillary retinal detachment; and stage 5, total retinal detachment, and retinal folds and holes.
